# Radiation Degradation of *β*-Glucan with a Potential for Reduction of Lipids and Glucose in the Blood of Mice

**DOI:** 10.3390/polym11060955

**Published:** 2019-06-01

**Authors:** Nguyen Thanh Long, Nguyen Thi Ngoc Anh, Bach Long Giang, Hoang Nghia Son, Le Quang Luan

**Affiliations:** 1 Nha Trang Vaccines and Biological Products Joint-Stock Company, Khanh Hoa, Viet Nam; ngthanhlong.biopharco@gmail.com; 2 Graduate University of Science and Technology, Vietnam Academy of Science and Technology, Ha Noi, Viet Nam; 3 Biotechnology Center of Ho Chi Minh City, Ho Chi Minh City, Viet Nam; ntngocanh09@gmail.com; 4 Nguyen Tat Thanh University, Ho Chi Minh City, Viet Nam; blgiang@ntt.edu.vn; 5 Institute of Tropical Biology, Vietnam Academy of Science and Technology, Ho Chi Minh City, Viet Nam; hoangnghiason@itb.ac.vn; 6 Hochiminh University of Natural Resource and Environment, Ho Chi Minh City, Viet Nam

**Keywords:** degradation, irradiation, low molecular weight β-glucan, water-soluble β-glucan, diabetic, dyslipidemia

## Abstract

Water-soluble and low molecular weight (Mw) *β*-glucans were successfully prepared by γ-irradiation of water-insoluble yeast *β*-glucans. The radiation dose used for the degradation of yeast *β*-glucan was remarkably reduced by increasing the pH of the sample or combining with hydrogen peroxide treatment. Radiation-degraded *β*-glucans with molecular weights in the range of 11−48 kDa reduced the total cholesterol, triglyceride, low density lipoprotein (LDL) cholesterol, and glucose levels in the blood of administered mice. The decreasing levels of both lipid and glucose indexes in the blood of tested mice strongly depended on the molecular weight of the *β*-glucan, and the radiation-degraded *β*-glucan with a molecular weight of about 25 kDa was found to be the most effective for the reduction of blood lipid and glucose levels. Particularly, the oral administration of 25 kDa *β*-glucan, with a daily dose of about 2 mg per head, reduced the total cholesterol, triglyceride, LDL-cholesterol, and glucose levels in the blood of tested mice to about 47.4%, 48.5%, 45.7%, and 47.2%, respectively. The effects on the reduction of blood lipid and glucose levels were also found to be stable after 20 days of stopping administration. These results indicate that the degraded *β*-glucan with a molecular weight of about 25 kDa prepared by γ-ray irradiation is a very promising ingredient that can be used in nutraceutical food for therapeutics of diabetic and dyslipidemia.

## 1. Introduction

*β*-glucans are non-starchy polysaccharides of *D*-glucose monomers, linked through *β*-glycosidic bonds with a long polymer chain. These natural polymers can be found in the cell walls of various natural sources, such as yeast, bacteria, seaweeds, cereals, legumes, and from many medicinal mushrooms [1,2,3,4]. *β*-glucans derived from cereals are polysaccharides of glucose residues with *β*(1→3) and *β*(1→4) linkages, while (1→3, 1→6)-*β*-glucans are mainly extracted from mushrooms and yeast cell walls [5]. Among these, *β*-glucans produced from *Saccharomyces cerevisiae* were reported to have more biological activities than *β*-glucans prepared from other sources [5]. Yeast *β*-glucans have been widely used in the food industry [6,7]. The well-known and specific bioactivities of this polymer are the enhancement of the host’s immune functions, which leads to antitumor and antimicrobial activities. *β*-glucan isolated from yeast has been demonstrated to stimulate responses, tolerance to oral antigens, and antimicrobial activity in animals [8,9,10,11,12]. Furthermore, *β*-glucan has been proven to have the ability to reduce glucose absorption in diabetic animals [13,14], prevent allergic reactions in mice [15], and provide hematoprotection against acetaminophen-induced toxicity in mice [16]. According to Sima et al. [17], both insoluble and soluble forms of this polymer are able to interact with lipids and biliary salts in the bowel, and consequently reduce cholesterol levels. Therefore, they may be developed to be a suitable therapeutic option for treating patients with dyslipidemia. 

Zlatkovic et al. [18], reported that the *β*-glucan extracted from cell walls of *Saccharomyces cerevisiae* consists of a common structure, with primary backbone chains of (1→3)-linked *β*-glucopyranosyl units (approximately 1,500 (1→3)-*β*-D-glucose units based), along which are randomly dispersed *β*-(1→6)-linked side chains. Therefore, this (1→3, 1→6)-*β*-glucan is usually a highly branched and water-insoluble polysaccharide [19]. Although yeast *β*-glucan has been proven to have more biological activities than *β*-glucan prepared form other source [5], the high molecular weight (Mw) and water-insoluble properties of yeast *β*-glucan are major disadvantages for the application of these novel polymers. Recently, Sung et al. [15,20] reported that degraded products of this natural polysaccharide with low molecular weight displayed stronger stimulation effects on immune activity in mice than those of native products with high molecular weight. In addition, Methacanon et al. [21] reported that the efficacy of β-glucan as an interleukin-8 inducer is associated with its molecular weight, and the degradation β-glucans demonstrated higher activity in interleukin-8 production compared to non-degraded samples. Because the high molecular weight and water-insoluble properties of native β-glucans cause some problems, such as high viscosity and low permeability into cells, degradation for the induction of a low molecular weight and water-soluble product not only solves the above barriers for application, but also increases the bioactivities of this novel biopolymer. 

So far, the treatments by acid [22,23] and hydrolysis by enzymes [24,25] have been widely applied for the degradation of polysaccharides, including *β*-glucan. Low molecular weight *β*-glucan products have been successfully prepared by chemical hydrolyses or enzymatic digestion [26,27,28,29], but some disadvantages, such as product purification, waste treatment, and high processing cost, still remain [30,31]. Gamma irradiation has been proposed as a useful method for the degradation of natural polysaccharides (cellulose, alginate, chitosan, etc.) via the cleaving of glycosidic bonds of polymer molecules [32,33,34]. The main advantages of radiation degradation for natural polysaccharides include the ability to forego chemicals or special conditions, ease of process control and large-scale application, a simple process, high degradation yield, high purity product, and low cost [32]. 

In addition, the synergistic degradation method using of γ-irradiation in combination with hydrogen peroxide treatment has been found to be a very efficient way for preparing low Mw polysaccharides, such as alginate and chitosan, with a low dose [35,36,37]. 

Although this method is unique and convenient, research on the degradation of *β*-glucan by ionizing radiation and in combination with hydrogen peroxide is still limited. 

The aim of the present study is to apply the γ-ray irradiation method for the degradation of *β*-glucan extracted from brewer’s yeast to prepare a water-soluble and low molecular weight product with high effectiveness for the reduction of lipid and glucose levels in blood of mice.

## 2. Materials and Methods

### 2.1. Materials

Water-insoluble *β*-glucan was extracted from the cell walls of brewer’s yeast, collected from Saigon-Binhduong Brewery (Binhduong province, Vietnam), Saigon-Binhtay Joint stock Company, by the method of William et al. [38], and the final product contained about 92% β-glucan (analyzed by a a 1,3:1,6 β-glucan (Yeast, mushroom) assay kit (K-YBGL), Megazyme Co., Wicklow, Ireland). The four-week-old Swiss mice used the for study were supplied by the Pasteur Institute in Ho Chi Minh City. All chemicals and reagents were purchased from Sigma Chemical Co. (St. Louis, MO, USA).

### 2.2. Degradation of β-glucan

In this study, the synergistic degradation method of γ-irradiation was used in combination with hydrogen peroxide treatment for increasing the degradation effect of *β*-glucan. For the degradation of *β*-glucan by radiation, 10 g *β*-glucan powder was suspended in 100 mL of deionized water, or 5 g *β*-glucan powder was suspended in 100 mL 1% hydrogen peroxide solution, for swelling overnight at room temperature, and then stirred for three hours. The pH of the mixtures was adjusted to 3, 5, 7, and 9 by NaOH 2 N before being exposed to a Co-60 irradiator for degradation at a dose up to 300 kGy and a dose rate of 3 kGy h^−1^. After irradiation, samples were centrifuged at 4500× *g* for 20 min to collect the supernatants. The supernatants were precipitated in ethanol and then centrifuged at 4500× *g* for 20 min to receive the solid parts. The precipitations were then washed with ethanol (80%) three times before being freeze-dried. The lyophilized samples were analyzed by a K-YBGL Kit for confirming the main content in the degraded β-glucan samples, before being stored at 4 °C for further experiments.

### 2.3. Determination of Water-Solubility in Irradiated β-glucan Samples

The water solubility of irradiated *β*-glucan was determined by the method described by Byun et al. [39]. After irradiation, the irradiated samples were first freeze-dried. Then, 5 g of freeze dried irradiated samples were put into a 50 mL glass tube with 25 mL of deionized water. The tube was then capped, before being vortexed for 20 min and centrifuged at 4500× *g* for 20 min. The supernatant was collected and then freeze dried to obtain the dried weight of water-soluble *β*-glucan products in the supernatant. Water solubility was calculated as follows: water solubility (%) = 100 × (weight of dried supernatant)/(weight of initial irradiated *β*-glucan powder).

### 2.4. Mw Determination 

The irradiated samples were put into a 50 mL glass tube and vortexed for 20 min before being centrifuged at 4500× *g* for 20 min. The supernatant was collected and freeze dried to collect the water-soluble *β*-glucan products. The changes in Mw of the irradiated *β*-glucan samples were analysed by a gel permeation chromatography (GPC) using an Agilent 1100 GPC system (Santa Clara, CA, USA) equipped with a bin pump (G1312A). Ultrahydrogel column models 250 and 500 from Waters (Milford, MA, USA) (7.8 id × 300 mm), equipped with a guard ultrahydrogel column (Waters, Milford, MA, USA), were used for monitoring. A volume of 20 µL of 0.1% *β*-glucan solution was loaded into the GPC system. Samples were processed at 40 °C and eluted with distilled water at a flow rate of 1.0 mL min^−1^ and monitored by a reflected index detector (RID, G1362A). 

### 2.5. FTIR Measurement

Fourier transform infrared (FTIR) spectroscopy of *β*-glucan samples was performed at ambient temperature using a Shimadzu FTIR-8100A spectrophotometer linked with a Shimadzu DR-8030 computer system (Shimadzu, Kyoto, Japan). Samples were prepared in a KBr pellet, formed by well dried mixtures containing 1% fine powder of *β*-glucan samples. All obtained spectra were the results of 128 scans at a resolution of 4 cm^−1^, in a wavelength range between 4000 and 400 cm^−1^.

### 2.6. ^1^H and ^13^C-NMR Spectrometry

^1^H and ^13^C-NMR spectra of degraded *β*-glucan were performed on a Fourier transformation nuclear magnetic resonance (NMR) spectrometer (Utrashield 500 plus, Brucker Bioscience Corporation, Billerica, MA, USA). The water-soluble *β*-glucan with a molecular weight of about 25 kDa was dissolved in D_2_O (Cambridge Isotope Laboratories, Inc., Tewksbury, MA, USA) at a concentration of 5 mg L^−1^. The ^1^H and ^13^C spectra were recorded at 500 MHz for ^1^H and 125 MHz for ^13^C under proton decoupling conditions with 10,000 scans.

### 2.7. X-ray Diffraction

Powder XRD data were collected at room temperature on the XRD D8 Advance Eco (Bruker, Billerica, MA, USA) with parafocusing Bragg–Brentano geometry, using CuKα radiation (lq = 1.5406 Å, U = 40 kV, I = 25 mA). Data were scanned with the ultrafast detector X’Celerator or with a scintillator detector equipped with a secondary curved monochromator over the angular range 10°–60° (2 theta), with a step size of 0.05° (2 theta) and a counting time of 0.5 s step^−1^. Data were evaluated by the software Diffrac.Eva V.4.3.1. (Madison, WI, USA) 

### 2.8. Experimental Design on Mice

For testing the effect of molecular weight of irradiated *β*-glucan on the reduction of blood lipid and glucose levels in mice, the mice, who were four weeks old and had an average body weight of about 20 g per head, were divided into three groups. Group Ia mice were served with normal feed (the checking group). The group IIa mice received fatty feed for eight weeks and were then supplied with only deionized water (the control mice group). The mice in group IIIa received fatty feed for eight weeks before being orally supplied with 2 mg per head daily of unirradiated *β*-glucan (molecular weight higher than 64 kDa) and irradiated *β*-glucan (molecular weight about 48, 25, and 11 kDa) for 40 days, followed by 20 days of supplementation with only deionized water.

The test for investigating the effect of the concentration of irradiated *β*-glucan was designed in the same way as testing the effect of molecular weight, in which group Ib was the checking group, group IIb were the control mice, and group IIIb were orally supplied, daily, with 0, 1, 2, 3, and 4 mg per head of *β*-glucan, with a molecular weight about 25 kDa for 40 days, followed by 20 days of supplementation with only deionized water.

### 2.9. Blood Plasma Analysis

After 20, 40, and 60 days of supplementatin with *β*-glucan samples, 0.5 mL blood from the myocardium of each tested mouse was collected and put into tubes containing heparin for analysis. The collected blood was then centrifuged at 4500× *g* for 10 min to separate the serum. The obtained serum was then used for analyzing the serum’s clinical chemistry indexes, including total cholesterol, triglyceride, low density lipoprotein (LDL) cholesterol, and glucose indexes by a BioSystem A17 analyzer (Brussels, Belgium). The net change of these indexes was the average mean difference in the blood of the mice before and after being administrated with deionized water or *β*-glucan samples.

All tests on the mice were conducted according to the ethical guidelines for animal research of the Institute of Tropical Biology, Vietnam Academy of Science and Technology, and followed the rules of the Declaration of Helsinki (ID: 49/QĐ-SHNĐ). All experiments were repeated three times. Data were statistically analyzed using the ANOVA test. The means were compared using the least significant difference (LSD) at a 5% probability level, and the standard deviations were calculated. 

## 3. Results and Discussion

### 3.1. Change in Mw and Water-Solubility of β-glucan by Irradiation

**Figure 1:**
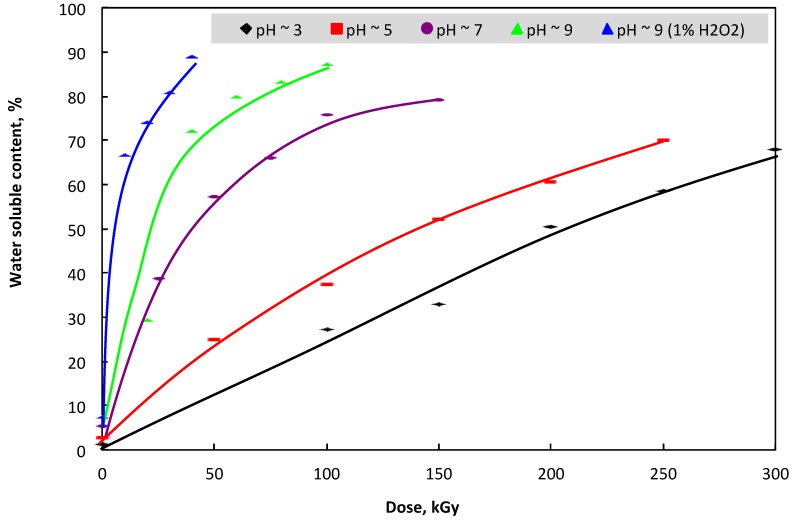
The increase in water-soluble content in *β*-glucan by gamma irradiation at various conditions.

The results in Figure 1 show that the reduction of water-soluble *β*-glucan molecular weight in the irradiated sample was proportional to the increase of absorbed dose. Therefore, it can be seen that the dose played a key role in the reduction of *β*-glucan molecular weight. In addition, Figure 1 also indicates that an increase in the pH of the irradiated sample led to a faster degradation of *β*-glucan Mw and the irradiation dose required for the degradation of *β*-glucan was much reduced. In particular, to reduce the molecular weight of the *β*-glucan sample to about 25 kDa, the required doses for irradiation of *β*-glucan samples in pH conditions of about 3, 5, 7, and 9 were found to be 200, 150, 75, and 50 kGy, respectively. This may be due to the swelling degree of *β*-glucan, which was increased with the increase in the pH of the sample, leading to an easier scission during irradiation. These results are in good agreement with those of Byun et al. [39] and Methacanon et al. [21], who reported that gamma irradiation could cause a decrease in the molecular weight of *β*-glucan in solutions.

**Figure 2:**
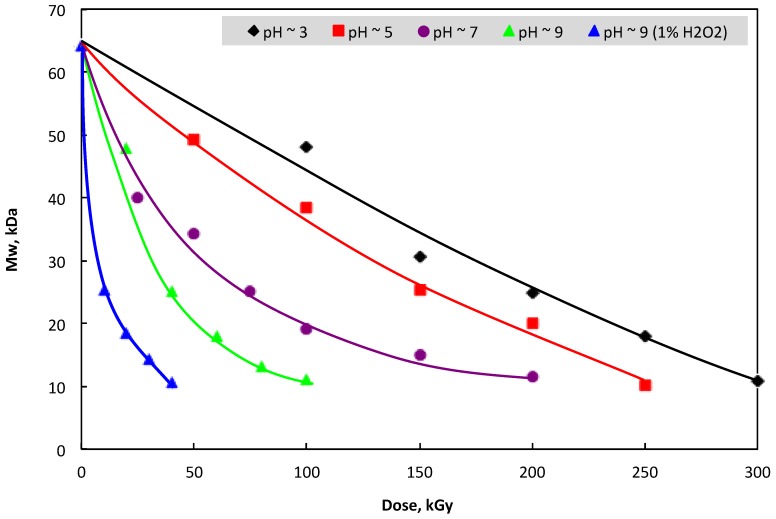
Change in molecular weight (Mw) of *β*-glucan by gamma irradiation at various conditions.

On the other hand, the *β*-glucan product derived from yeast cell walls was not soluble in water, and this was an impediment for utilization of this polymer. So far, the modifications of its structure by carboxylmethylation, phosphorylation, and sulfation have been reported as the main methods for the induction of water-soluble *β*-glucan [26,38,40]. Additionally, the reduction of the molecular weight of this polymer by degradation is another way for preparing the water-soluble *β*-glucan. Ershos [41] reported that in aqueous solution, the radiation degradation of polysaccharides is shown by a decrease in molecular weight, while Byun et al. [39] confirmed that the degradation of *β*-glucan molecular weight led to an increase in the water-soluble content of the irradiated sample. In this study, the results in Figure 2 suggest that the water-soluble content in the irradiated samples increased with the increase of irradiation dose. This may be due to the increase of degradation in the chains of *β*-glucan molecules with the increase of irradiation dose, as shown in Figure 1. These results are in good agreement with those reported by Byun et al. [39]. On the other hand, the pH conditions in the sample also played an important role for the degradation of *β*-glucan’s molecular weight. In particular, the water-soluble contents were 66.7, 69.0, 78.3, 86.3, and 87.9% in samples irradiated at 300 kGy in pH ~ 3, 250 kGy with pH ~ 5, 150 kGy with pH ~ 7, 100 kGy with pH ~ 9, and 10 kGy with pH ~ 9 in combination with 1% H_2_O_2_, respectively. The results from Figure 1 and Figure 2 also revealed clearly that the increase in the reduction rate of *β*-glucan’s molecular weight with the increase in the pH of the irradiated samples was the main reason for enhancing the soluble content in irradiated samples with high pH conditions.

**Figure 3:**
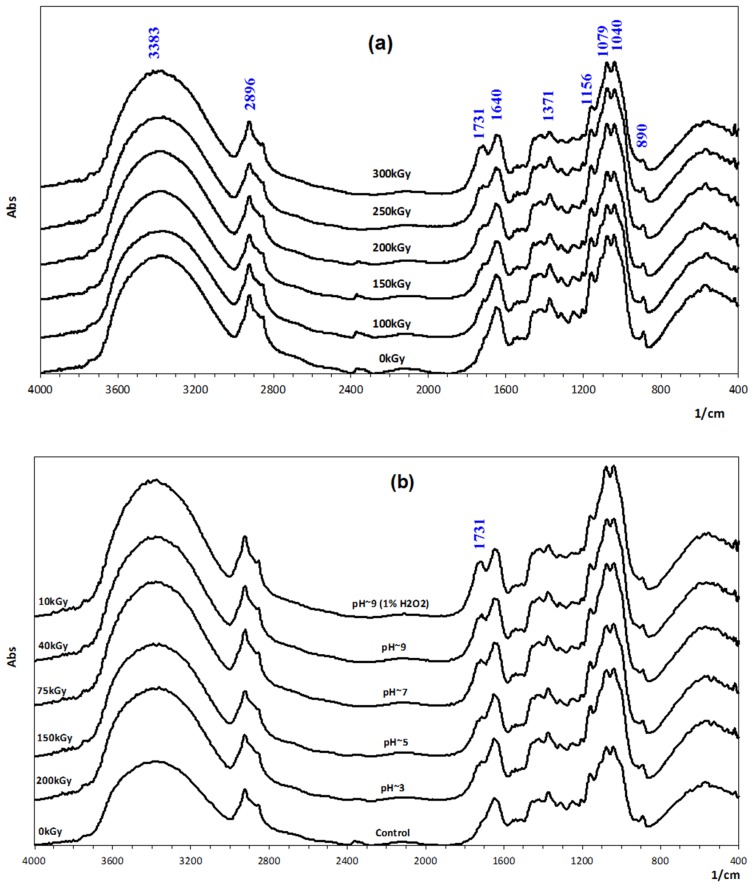
The Fourier transform infrared (FTIR) spectra of *β*-glucan samples irradiated at different doses (**a**) and in various pH conditions, with and without the present of H_2_O_2_ (**b**).

**Figure 4:**
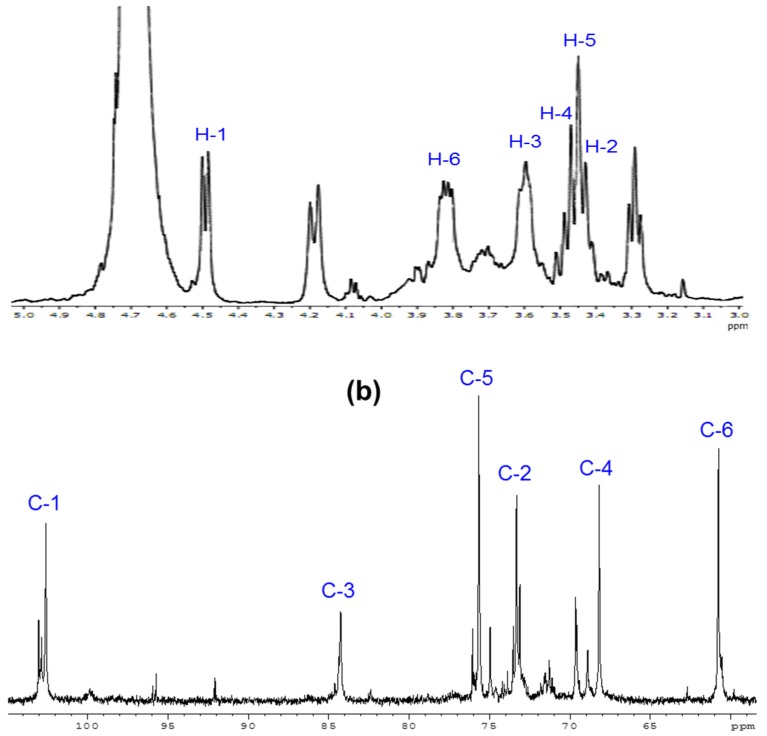
The ^1^H (**a**) and ^13^C nuclear magnetic resonance (NMR) (**b**) spectra of degraded *β*-glucan with molecular weight of about 25 kDa.

In addition, the structural characteristics of unirradiated and irradiated samples were also analysed by FTIR spectroscopy. The results in Figure 3a (spectra of *β*-glucan samples irradiated at different doses) and Figure 4b (spectra of *β*-glucan samples irradiated at various pH conditions and in combination with 1% H_2_O_2_ treatment) indicate that the strong bands assigned to the O–H and C–H stretching vibrations of the polysaccharide were recorded at 3383 and 2896 cm^−1^, respectively. The peak at 1640 cm^−1^ is presumably a feature of tightly bound water in the biopolymers [21], while the band at 1371 cm^−1^ was attributed to the CH_2_. Bands in the region of 1156, 1079, and 1040 cm^−1^ were assigned to C–C, C–OH, and C–O–C linkages (glycosidic bonds), respectively [42]. The peak corresponded to β-D-glucan found at 890 cm^−1^. It can be seen that all the above mentioned bands in the spectra of samples irradiated in various conditions (various doses and different pH values and in 1% H_2_O_2_) are almost no different from those in the spectrum of the native sample. Exceptionally, the peak appearing at 1731 cm^−1^ linkage was only a weak shoulder in the spectrum of the native sample, but the intensity of this peak increased with the increase in the irradiation doses, the increase in the pH value of the samples, and the addition of H_2_O_2_. This peak may be assigned for C=O linkage in the end group of the β-glucan molecule (the open-chain form of the end glucose unit) after scission. Therefore, the number of end groups increased with the decrease in the Mw of the degraded samples, leading to the increase in the peak intensity in the spectra of the degraded samples. Several papers, which reported on the degradation of cellulose and its derivatives by γ-ray irradiation, proved that the characteristic effect of radiation on natural polysaccharides, whatever the conditions, was mainly degradation, due to the scission of glycosidic bonds by radiation. In an aqueous solution, the radicals (^•^OH) from irradiated water must be responsible for the degradation of polysaccharides [41,43,44] and polysaccharides are typical degradable materials under ionizing radiation through the glycosidic bonds cleavage, resulting in the reduction in their molecular weights. The main mechanism for the degradation of polysaccharides by radiation can be described in the following equation:

R−H + ^•^OH **→** R^•^ + H_2_O **→** F^•^_1_ + F_2_ (scission).

In general, hydroxyl radical reacts with polysaccharide macromolecules (R−H) exceedingly rapidly for abstracting a C-bonded H atom and generating R^•^ radicals. These radicals then undergo further reactions to generate lower molecular weight fragments of the main chain after scission (F^•^_1_, F_2_), before ending up.

Thus, hydroxyl radicals are powerful oxidizing species which can attack the β-glycosidic bonds of polysaccharides. Hence, the radiation treatment on polysaccharides in the presence of hydrogen peroxide could reduce its molecular weight very effectively, and the primary reactions might further occur as follows:

$$H_{2}O\overset{\mathrm{hv}}{\to }H_{2}{,H}_{2}O_{2}{,e}^{-}{}_{\mathrm{aq}}{,H}^{•}{,\;\mathrm{OH}}^{•}{,\;H}_{3}O^{+};$$

$$H^{•}{+\;H}_{2}O_{2}\overset{\mathrm{hv}}{\to }H_{2}{O\;+\;\mathrm{OH}}^{•};$$

$$e^{-}{}_{\mathrm{aq}}{+\;H}_{2}O_{2}\overset{\mathrm{hv}}{\to }{\mathrm{OH}}^{•}{+\;\mathrm{OH}}^{-};$$

$$H_{2}O_{2}\overset{}{\to }{2\mathrm{OH}}^{•};$$

$${R-H\;+\;\mathrm{OH}}^{•}\to R^{•}{+\;H}_{2}O;$$

$$R^{•}\to F_{1}{+\;F}_{2}.$$

The above results indicate that γ-ray irradiation is considered an efficient method for the degradation of water-insoluble *β*-glucan extracted from yeast cell walls. For the degradation of this natural polymer by γ-rays, the •OH radicals were responsible for degradation by scission at glycosidic bonds in *β*-glucan molecules. The irradiation mainly caused the scission at glycosidic bonds in the structure of *β*-glucan, leading to the reduction in the degree of polymerization, and the degradation rate in the high pH condition was higher than that in low pH condition, which reduced the required dose for degradation. Notably, the combined treatment with H_2_O_2_ strongly reduced the radiation dose for the degradation of the *β*-glucan sample. These results are in good agreement with previous reports [35,36,37].

In addition, the ^1^H and ^13^C-NMR spectra of degraded *β*-glucan with a molecular weight of about 25 kDa, presented in Figure 4, indicate the structure by signals for the protons and carbon atoms of the glucose unit of the main chain of yeast β-glucan. Particularly, the chemical ships observed at 4.48, 3.43, 3.59, 3.45, 3.47, and 3.82 ppm (Figure 4a) were assigned for H-1, H-2, H-3, H-4, H-5, and H-6, respectively, while chemical shifts corresponding to C-1, C-2, C-3, C-4, C-5, and C-6 (Figure 4a) appeared at 102.58, 73.32, 84.25, 68.16, 75.66, and 60.75 ppm, respectively. The assignment of all signals is in accordance with published spectra [21,39,45,46].

**Figure 5:**
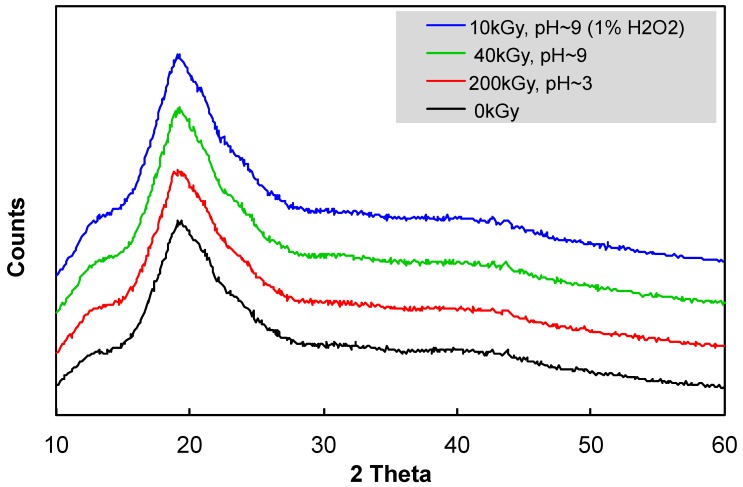
XRD curves of *β*-glucan samples irradiated at different doses in various pH conditions, with and without the present of H_2_O_2_.

The crystallinity and its changes with irradiation in different conditions were also observed by XRD. It can be seen clearly from Figure 5 that all samples were almost homogenous, and no clear evidence of a crystalline peak was observed within the range of 10–60° (2 theta). Only a very slight crystallinity peak appeared around 42.3° (2 theta). Thus, the γ-ray irradiation of *β*-glucan in different pH conditions and in combined treatment with H_2_O_2_ did not cause any change to the crystallinity in the structure of the sample.

### 3.2. Effect of Irradiated β-glucan on the Reduction of the Lipid and Glucose Index in the Blood of Mice

#### 3.2.1. Effect of β-glucan Mw 

**Figure 6:**
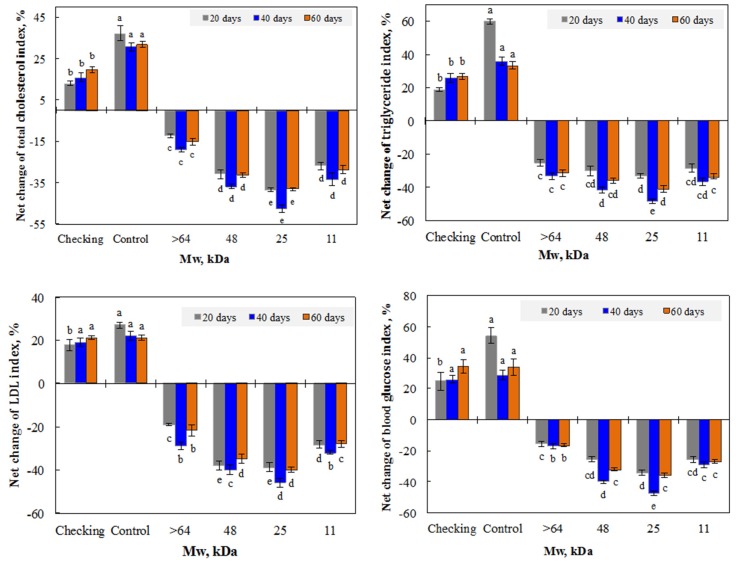
Net change of blood lipid and glucose indexes of mice after 20 and 40 days administrating with unirradiated and irradiated *β*-glucan samples, and after stopping administration for the following 20 days (60 days).

Hypercholesterolemia and diabetes are risk factors of cardiovascular disease. Both insoluble and soluble *β*-glucans have been proven to have an ability to reduce cholesterol levels by interacting with lipids and biliary salts in the bowel [16]. This natural polymer has also been demonstrated to reduce glucose absorption in diabetic animals [13,14]. The bioactivities of *β*-glucans were reported to depend on their molecular weight and solubility. In this study, unirradiated chitosan and irradiated *β*-glucans with a molecular weight of approximately 11, 25, and 48 kDa were used for testing their effects on the reduction of the blood lipid and glucose index after oral administration in mice. It can be seen from Figure 6 that after 20 days of being administrated with both unirradiated and irradiated *β*-glucan samples, the indexes of lipid and glucose in the blood of treated mice were significantly reduced. In the case of mice administrated with irradiated *β*-glucan, the total cholesterol, LDL cholesterol, triglyceride, and glucose indexes were strongly reduced, by 27.0%–30.8%, 28.3%–37.9%, 28.6%–30.2%, and 25.5%–34.1%, respectively, while the reduction of these indexes in the blood of mice administrated with unirradiated *β*-glucan were found to be about 12.4%, 19.2%, 25.3%, and 15.6%, respectively. These results indicate that compared to unirradiated *β*-glucan, irradiated *β*-glucans with a molecular weight from 11 to 48 kDa had a stronger effect on the reduction of blood lipid and glucose in mice. Among irradiated samples, the irradiated *β*-glucan with a molecular weight of about 25 kDa displayed the strongest effect for the reduction of all tested indexes.

The results in Figure 6 also point out that the further 20 days supplementation of *β*-glucan samples continued reducing blood lipid and glucose indexes in treated mice (group IIIa). After 40 days of administration, the blood glucose in mice supplemented with unirradiated *β*-glucan reduced by only 16.9%, while the reduction of this value in mice supplemented with irradiated *β*-glucan samples reduced by about 29–47.3%. The stronger reductions of blood total cholesterol (33.5–47.2%), LDL cholesterol (31.9%–45.7%), and triglyceride (36.8%–45.5%) were also found in the group of mice supplemented with irradiated *β*-glucans, while these indexes were slightly increased in the blood of the checking mice dieted with normal feed (class Ia) and slightly decreased in blood of the control mice dieted with fatty feed (group IIa). After administration for 40 days, the mice in group IIIa were no longer supplemented with *β*-glucan samples, for evaluation of the post effect, the results in the following 20 days (after 60 days) showed that there was a slight increase in all tested indexes in the blood of mice administrated with *β*-glucans, but these levels were still far lower than those in the blood of mice in groups Ia and IIa. These results assert that *β*-glucans had a post effect for keeping the blood lipid and glucose levels in a stable condition after stopping supplementation for 20 days. It can be seen from Figure 6 that the irradiated *β*-glucans with a low molecular weight and water-solubility showed a better effect on the reduction of blood lipid and glucose indexes in mice compared to the unirradiated sample. Recent reports have demonstrated that the administration of degraded *β*-glucan with low molecular weight (from 9 to 35 kDa) enhanced the immune activities, spleen cell proliferation, and Th1 cytokine production, in tested mice [17,20,47]. It is well known that the functional activities of *β*-glucans are influenced by its polymer length. The mechanism is still not clear, but this effect may be due to the molecular weight. In this study, the irradiated *β*-glucan with a molecular weight of about 25 kDa displayed the highest activity for the reduction of both blood lipid and glucose indexes. This may also be due to the appropriate molecular weight of the irradiated *β*-glucan. According to Waszkiewicz-Robak, the low molecular weight soluble β-glucans can uptake easier and then inhibit cholesterol synthesis in the liver, leading to the reduction of the cholesterol level in blood [48]. The *β*-glucan with a molecular weight of about 25 kDa was selected for investigating the suitable dose for oral administration in the following experiment.

#### 3.2.2. Effect of Irradiated β-glucan Concentration 

**Figure 7:**
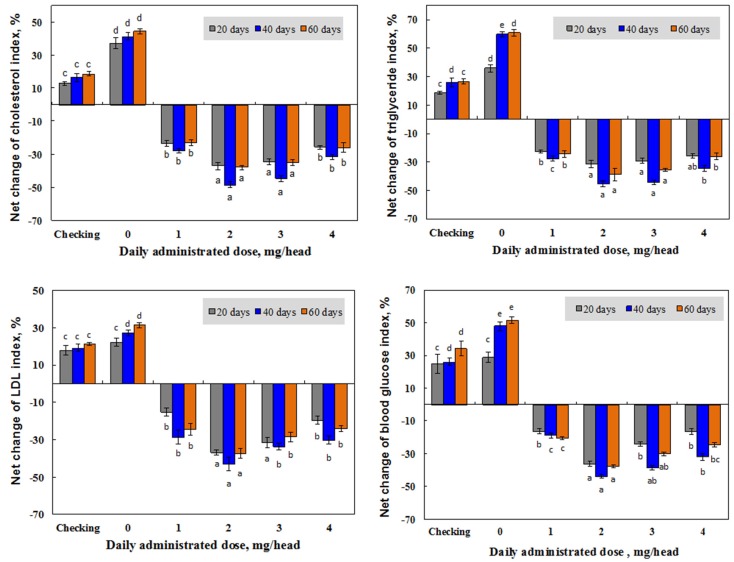
Net change of blood lipid and glucose levels in mice after 20 and 40 days administration of *β*-glucan with a molecular weight of about 25 kDa at different doses, and after stopping administrating for following 20 days (60 days).

Several papers have reported the effect of concentration for dieting with *β*-glucans. According to th U.S. Food and Drug Administration, 3 g of *β*-glucan should be consumed daily to attain a clinically relevant decrease in total cholesterol levels, and food products must contain at least 0.75 g of *β*-glucan per serving [49]. In addition, the meta-analysis of Talati et al. [50] proved that a median *β*-glucan dose of 7 g/day could reduce LDL cholesterol levels by 0.26 mM L^−1^, while another meta-analysis of AbuMweis et al. [51] demonstrated that LDL cholesterol was lowered by 0.27 mM L^−1^ following a median *β*-glucan dose of 5 g/day. In a comparison of administration methods of four *β*-glucan products extracted from different sources in mice, Vetvicka and Vetvickova [52] reported that the individual product extracted from yeast cell walls was the best for immune-stimulating activities, and these activities in mice administrated with an intraperitoneal injection was slightly lower than those in mice supplemented by oral application. These authors also concluded that the somehow lower effects after oral stimulation could be easily overcome by repeated oral doses. In this study, the daily doses for oral administration of *β*-glucan with a molecular weight of about 25 kDa were conducted at 1, 2, 3, and 4 mg per mouse in order to find a suitable dose for the application of this novel product. The results in Figure 7 show that after 20 daily supplementation doses of 1–4 mg, the reductions in the blood total cholesterol, LDL cholesterol, triglyceride, and glucose levels in mice administrated by *β*-glucan with a molecular weight of about 25 kDa (group IIIb) were found to be 23.6–37.1%, 15.4–36.9%, 22.7–31.5%, and 16.4–36.2%, respectively. These levels were found to be increased in the blood of mice in the checking group (group Ib) and control group (group IIb). The analytic results also demonstrated that the levels of these indexes were continuously reduced in supplemented mice, and the daily supplemented dose of 2 mg displayed the strongest effect on the reduction of blood total cholesterol (48.4%), LDL cholesterol (43.2%), triglyceride (45.4%), and glucose (43.9%). *β*-glucans have also been reported to have ability to reduce blood cholesterol (total and LDL cholesterol) and glucose levels, but the detailed mechanisms of this action are still unclear. Most researchers mentioned that *β*-glucan may play a major role in reducing the absorption of glucose and cholesterol, as well as in inhibiting the reabsorption of bile salts in the small intestine [53,54,55]. In the present study, the irradiated *β*-glucan with a low molecular weight (25 kDa) and water-soluble properties also showed strong effects on the reduction of blood lipid and glucose in tested mice, and this *β*-glucan may also play an important role in reducing the absorption of glucose and cholesterol in supplemented mice. The post effect of the *β*-glucan product with a molecular weight of about 25 kDa was also investigated and the results in Figure 7 indicate that after ceasing administrating the *β*-glucan sample with a molecular weight of about 25 kDa, the levels of lipid and glucose in supplemented mice were slightly increased, but they were still far lower than those in mice of the control group (group IIb) and checking group (group Ib). The strongest reduction changes for the levels of total cholesterol (38.1%), LDL cholesterol (37.3%), triglyceride (38.9%), and glucose (37.7%) were found in mice supplemented with a daily dose of 2 mg *β*-glucan with a molecular weight of about 25 kDa. These results are in good agreement with the report of Sung et al. [20] that the effective administration dose of low molecular weight *β*-glucan for the prevention of allergic reactions in mice was about 125 mg kg^−1^ body weight (equivalent to 2.5 mg per a mouse with 20 g body weight).

## 4. Conclusions

In conclusion, γ-ray irradiation is a useful method for the degradation and induction of low Mw and water-soluble *β*-glucan. The irradiation did not cause any change to the basic structure of the *β*-glucan products after irradiation, except the reduction in the degree of polymerization and the degradation rate in the high pH condition was higher than that in low pH condition, which reduced the required dose for degradation. The irradiated *β*-glucan had a stronger effect on the reduction of lipid and glucose indexes than that of unirradiated *β*-glucan. The present study also clearly showed that the irradiated *β*-glucan with a molecular weight of about 25 kDa displayed the strongest reduction for the levels of blood total cholesterol (48.4%), LDL cholesterol (43.2%), triglyceride (45.4%), and glucose (43.9%) after 40 days administration with a daily dose of 2 mg. The water-soluble *β*-glucan with a molecular weight of about 25 kDa prepared by the irradiation method may be considered a functional ingredient in nutraceutical food production for therapeutics of diabetic and dyslipidemia.

## Abbreviations

ANOVA	Analysis of variance
Da	Dalton
GPC	Gel permeation chromatography
Gy	Grey
FTIR	Fourier transform infrared spectroscopy
LDL	low density lipoprotein
Mw	molecular weight
